# The passage of cells can improve the detection rate of avian leukosis virus to facilitate the elimination of avian leukosis in chickens

**DOI:** 10.1186/2193-1801-2-138

**Published:** 2013-03-29

**Authors:** Xiuzhen Wang, Bo Wang, Peipei Zhang, Hegang Cheng, Shuhong Sun

**Affiliations:** College of Veterinary Medicine, Shandong Agricultural University, Taian, Shandong PR China

**Keywords:** Avian leukosis virus, Cells passage, Detection rate of p27 antigen

## Abstract

Avian leukosis (AL) is one of the most harmful diseases to the poultry industry in China. The detection of the avian leukosis virus (ALV) p27 antigen plays a decisive role in the elimination of avian leukosis. To explore the influence of passaging cells on the detection rate of the ALV p27 antigen, 21 aseptic anticoagulated blood samples were collected from 21 chickens for which the cloacal swabs were positive for the p27 antigen to inoculate two sets of cell culture plates containing DF1 cells. The cells were cultured for 4 d, one set was passaged, and the other set was not. After the DF1 cells had been cultured for 9 d, the ALV p27 antigen in the supernatants of the two sets was detected by ELISA. The results showed that the p27 antigen-positive rate for the passaged cells was 71.43% (15/21), higher than that of the cells that were directly cultured, which was 42.86%. There was a strong correlation, as high as 0.928, with respect to the S/P value of the p27 antigen in the supernatant between the two sets. In conclusion, there was a strong correlation between the results for the passaged and unpassaged cells, and the passage of cells greatly improved the detection of the p27 antigen.

## Introduction

Avian leukosis (AL) is caused by the avian leukosis virus (ALV), which is associated with a variety of malignant neoplasms, including lymphoid and myeloid leukosis, and other production problems in chickens (Witter, 1997). AL is found in most countries that have large-scale farms and is one of the most harmful diseases in the poultry industry, causing serious economic losses (Sun and Cui 2007; Zhang et al. 2011). The ALV p27 gene is a highly conserved gene among the different subtypes of ALV, and the p27 antigen is a serogroup-specific antigen. This antigen can be detected by ELISA for many kinds of samples for the diagnosis of avian leukosis, such as albumen, vaginal secretions, meconium, cloaca cotton swab, and also can be used to detect the propagation of the virus (Spencer et al. 1984; Spencer et al. 1976; De Boer et al. 1984).

At present, there are no effective vaccines or drugs to prevent or treat AL. Most countries control ALV primarily through the continual detection of the ALV p27 antigen and the elimination of antigen-positive chickens. Presently, there are three common schemes for eliminating ALV from breeding flocks: (1) Detect the p27 antigen using cloacal cotton swabs or albumen and eliminate the antigen-positive chickens. (2) Aseptically collect anticoagulated blood from the core group, use this blood to inoculate DF1 cells, and detect the p27 antigen after 9 d of culture. Then, detect the p27 antigen in the meconium of the next generation of chickens to continuously eliminate all antigen-positive chickens. This method is the international gold standard for AL elimination. (3) Combine the first and second methods to separate the p27 antigen-positive and negative chickens and aseptically collect anticoagulated blood or albumen from p27 antigen-positive chickens to inoculate DF1 cells for p27 antigen detection. Using this method, we can distinguish endogenous and exogenous ALV, just eliminating exogenous ALV-positive chickens and retaining the endogenous ALV-positive chickens (Spencer et al. 1984).

The collections of aseptic anticoagulated blood to inoculate DF1 cells and the detection of the p27 antigen in the supernatant after 9 d of culture play a vital role in the elimination of AL. This method complements the method of the direct detection of the p27 antigen in cloaca swabs or albumen. In this study, the influence of passaging cells on the detection rate and the titer of the ALV p27 antigen were investigated to explore a better method for timely and thorough AL elimination.

## Materials and methods

Twenty-one chickens for which cloacal cotton swabs were positive for the p27 antigen were numbered from 01 # to 21 #. An aseptic anticoagulated blood sample was collected from each chicken, and then the samples were centrifuged at 1500 rpm for 5 min to isolate the leukocytes. The leukocytes were then suspended in 200 ul and used to inoculate two sets of cell culture plates containing DF1 cells.

Each set of cell culture plates has 24 holes, with 21 holes inoculated and one hole inoculated with a blank to serve as the negative control, which was numbered 22#. Both the sets were dealt with uniformly. Thereafter, the inoculated DF1 cultures were incubated under CO2 at 37°C for 2 h, and 1% DMEM was substituted for the 5% DMEM during continuous culturing. After the cells had been cultured for 4 d, the cells of one set were passaged, and the cells in other set were not. The supernatants of both sets were collected for p27 antigen detection after 9 d of culturing.

All the experimental methods comply with current ethical consideration.

### The influence of cell passaging on the p27 antigen-positive rate of the supernatant

After 9 d of culturing, the DF1 supernatant from each sample was collected for ALV p27 antigen detection in one reaction plate using the Avian Leukosis Virus Antigen Test Kit® (IDEXX, USA) according to the manufacturer’s instructions, and the p27 antigen-positive rates of the passaged and unpassaged cells were compared.

### The influence of cell passaging on the titer of the p27 antigen in the supernatant

The S/P values determined using an ELISA for the p27 antigen in the 9-d supernatants of the passaged and unpassaged samples were compared, and the correlation was assessed using the matched-pair *T* test in SPSS 17.0.

## Results

### The influence of cell passaging on the p27 antigen-positive rate of the supernatant

The results show that cell passaging can improve the p27 antigen detection rate when using cell supernatant, as the data was shown (Table 1). The ALV p27 antigen-positive rate for the supernatant of the passaged cells was 71.43% (15/21), which was far higher than that for the supernatant of the unpassaged cells, 42.86% (9/21). All of the positive unpassaged samples except 18# were also positive for the passaged samples, and the negative control 22# was negative for p27 antigen.

**Table 1:** **The test results for the ALV p27 antigen in the 9-d cell supernatants**

Number	1	2	3	4	5	6	7	8	9	10	11	12	13	14	15	16	17	18	19	20	21	positive rate
Passaged sample	**-**	+	+	+	+	+	**-**	**-**	**-**	+	+	+	+	+	+	+	+	**-**	+	+	**-**	15/21
Unpassaged sample	**-**	+	+	**-**	**-**	+	**-**	**-**	**-**	**-**	**-**	+	**-**	+	+	**-**	**-**	+	+	+	**-**	9/21

### The influence of cell passaging on the titer of p27 antigen in the supernatant

**Figure 1:**
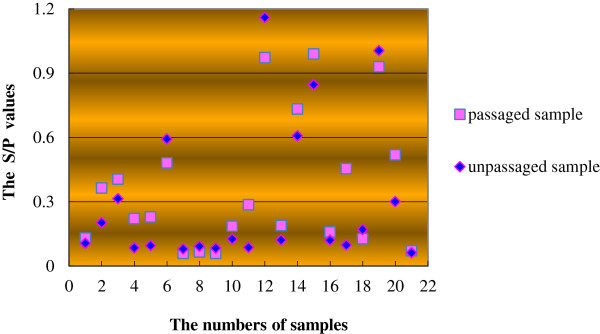
**The S/P values of the supernatant of cells cultured for 9 d.**

The S/P values of most of the passaged samples were higher than those of unpassaged samples. Only seven passaged samples had lower titers than the unpassaged samples, and these differences were small. When the S/P value of a sample was greater than 0.2, that sample was judged to be positive. The correlation coefficient of the S/P value between the passaged samples and the unpassaged samples was as high as 0.928. The results showed that there is a strong correlation between the passaged and unpassaged samples with respect to the S/P value, and the passaging of cells improved the p27 antigen titer in the 9 d culture supernatants (Figure 1).

## Discussion

ALV has spread all over the world since the early 20th century and has resulted in serious harm to the bird farming industry (Ochi et al. 2012). At present, the primary means to control the disease in most countries combines direct ALV p27 antigen detection with detection of the ALV p27 antigen in the supernatant of cells directly cultured for 9 d, followed by the elimination of all positive chickens. This process is continually repeated to eliminate infected chickens. In China, ALV has spread to native varieties of poultry and is a serious threat to the maintenance of high-quality varieties of chickens (Wang et al. 2011; Zhang et al. 2010; Cheng et al. 2005; Qi et al. 2011; Zhao et al. 2010; Qian et al. 2011). Therefore, it is important to remove all ALV p27 antigen-positive chickens from stock breeding groups as soon as possible.

Based on the ALV elimination scheme commonly used internationally for stock breeding flocks, this study explored the influence of passaging cells on the detection rate and titer of the ALV p27 antigen as part of a protocol for timely and thorough AL elimination. The results show that the passage of cells can improve the p27 antigen-positive detection rate in the 9-d cell culture supernatants, leading to a positivity rate of 71.43% (15/21), which was far higher than that of the unpassaged cells, 42.86% (9/21). In addition, all of the positive unpassaged samples except for 18# were positive for the passaged samples. There was a strong correlation between the passaged and unpassaged samples with respect to the S/P value, and the passage of cells improved the p27 antigen titer. These results suggest that, compared with the classic method in which the cells are cultured for 9 d without passaging, a protocol in which the cells are passaged in the middle of the culture period can identify more ALV p27 antigen-positive chickens in the same amount of time. Therefore, the passaging of cells can speed up the AL elimination process, which has positive practical results and objective economic significance.
